# Functional network dynamics revealed by EEG microstates reflect cognitive decline in amyotrophic lateral sclerosis

**DOI:** 10.1002/hbm.26536

**Published:** 2023-12-13

**Authors:** Marjorie Metzger, Stefan Dukic, Roisin McMackin, Eileen Giglia, Matthew Mitchell, Saroj Bista, Emmet Costello, Colm Peelo, Yasmine Tadjine, Vladyslav Sirenko, Serena Plaitano, Amina Coffey, Lara McManus, Adelais Farnell Sharp, Prabhav Mehra, Mark Heverin, Peter Bede, Muthuraman Muthuraman, Niall Pender, Orla Hardiman, Bahman Nasseroleslami

**Affiliations:** ^1^ Academic Unit of Neurology, School of Medicine, Trinity Biomedical Sciences Institute, Trinity College Dublin University of Dublin Dublin Ireland; ^2^ Department of Neurology, University Medical Centre Utrecht Brain Centre Utrecht University Utrecht The Netherlands; ^3^ Discipline of Physiology, School of Medicine, Trinity Biomedical Sciences Institute, Trinity College Dublin University of Dublin Dublin Ireland; ^4^ Neural Engineering with Signal Analytics and Artificial Intelligence, Department of Neurology University of Würzburg Würzburg Germany; ^5^ Department of Psychology Beaumont Hospital Dublin Ireland; ^6^ Department of Neurology Beaumont Hospital Dublin Ireland; ^7^ FutureNeuro ‐ SFI Research Centre for Chronic and Rare Neurological Diseases Royal College of Surgeons Dublin Ireland

**Keywords:** cognitive‐behavioural impairments, EEG microstates, motor neuron disease, neurodegeneration, resting‐state EEG, temporal dynamics, transition probabilities

## Abstract

Recent electroencephalography (EEG) studies have shown that patterns of brain activity can be used to differentiate amyotrophic lateral sclerosis (ALS) and control groups. These differences can be interrogated by examining EEG microstates, which are distinct, reoccurring topographies of the scalp's electrical potentials. Quantifying the temporal properties of the four canonical microstates can elucidate how the dynamics of functional brain networks are altered in neurological conditions. Here we have analysed the properties of microstates to detect and quantify signal‐based abnormality in ALS. High‐density resting‐state EEG data from 129 people with ALS and 78 HC were recorded longitudinally over a 24‐month period. EEG topographies were extracted at instances of peak global field power to identify four microstate classes (labelled A‐D) using K‐means clustering. Each EEG topography was retrospectively associated with a microstate class based on global map dissimilarity. Changes in microstate properties over the course of the disease were assessed in people with ALS and compared with changes in clinical scores. The topographies of microstate classes remained consistent across participants and conditions. Differences were observed in coverage, occurrence, duration, and transition probabilities between ALS and control groups. The duration of microstate class B and coverage of microstate class C correlated with lower limb functional decline. The transition probabilities A to D, C to B and C to B also correlated with cognitive decline (total ECAS) in those with cognitive and behavioural impairments. Microstate characteristics also significantly changed over the course of the disease. Examining the temporal dependencies in the sequences of microstates revealed that the symmetry and stationarity of transition matrices were increased in people with late‐stage ALS. These alterations in the properties of EEG microstates in ALS may reflect abnormalities within the sensory network and higher‐order networks. Microstate properties could also prospectively predict symptom progression in those with cognitive impairments.

## Graphical abstract

In amyotrophic lateral sclerosis, both static and dynamic properties of resting‐state EEG microstates were found to be disrupted, indicating abnormalities within the sensory network as well as higher‐order networks. These alterations in microstate properties hold the potential to serve as predictive indicators for symptom progression, particularly in individuals with cognitive impairments.

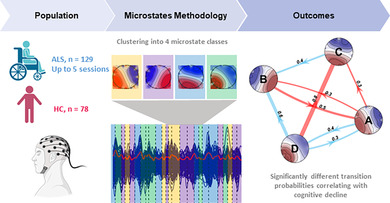

## 1 INTRODUCTION

Amyotrophic lateral sclerosis (ALS) is a neurodegenerative disease associated with progressive upper and lower motor neuron degeneration. ALS involves motor, cognitive and behavioural decline, and death typically occurs as a result of ventilatory failure within 3–5 years from first symptoms (Costello et al., 2021; Evans et al., 2015; Hardiman et al., 2017; Phukan et al., 2012). Up to 50% of people with ALS exhibit evidence of cognitive dysfunction and ~14% reach the threshold for ALS–frontotemporal dementia (FTD) diagnosis (Phukan et al., 2012). There is no effective treatment for ALS, and there remains an urgent need for cost‐effective, reliable biomarkers to quantitatively assess cognitive and motor decline.

Whole‐brain resting‐state electroencephalographic (EEG) studies can provide robust evidence of motor and extra‐motor degeneration in ALS. The most recent findings of frequency domain and source localisation analyses include increased co‐modulation in the fronto‐parietal area (θ, γ‐band), and decreased synchrony in the fronto‐temporal areas (δ, θ‐band) (Dukic et al., 2019; Nasseroleslami et al., 2019). Although abnormal functional connectivity in both sensor and source‐space has been shown, there is limited understanding of the temporal dynamics of brain networks in ALS.

Insights into the temporal dynamics of brain networks can be gained through analysing brain ‘microstates’. Microstates are defined as transient, quasi‐stable electric field configurations that repeat sequentially over time within an EEG recording. Microstate analysis involves identifying recurring topographical patterns of spontaneous neural activity across multiple time points and categorizing the EEG topography at each time point into one of these distinct microstate classes. Microstate transitions were originally attributed to changes in the coordination of synaptic activity (Lehmann et al., 1987). These distinct re‐occurring topographies of the scalp electrical potential (‘scalp maps’) have a duration spanning from milliseconds to seconds. Four canonical classes (labelled A–D) of microstates have been repeatedly described and have been associated with well‐established resting‐state networks (RSNs) in fMRI, based on the estimated brain regions generating each microstate (Michel & Koenig, 2018). Analysing these microstates allows us to investigate changes in the temporal dynamics *of* brain networks instead of changes in functional connectivity *between* networks, which is more typically examined in EEG studies (Gschwind et al., 2016).

Changes in the properties of microstates have been previously associated with altered states of consciousness (Bai et al., 2021; Bréchet & Michel, 2022; Zanesco et al., 2021) and with neurological or neuropsychiatric conditions (Al Zoubi et al., 2019; Dierks et al., 1997; Faber et al., 2021; Gschwind et al., 2015; Koenig et al., 1999; Michel & Koenig, 2018; Nishida et al., 2013). Alterations in microstate characteristics are thought to represent alterations in the rhythm of neural processes. However, it is the microstates' temporal dependencies that can perhaps give us the greatest insight into how brain function is altered in neurodegenerative diseases like ALS. Neurological conditions seem to alter the brain's functional resting state transitions; forcing the brain to stay and/or change to specific functional networks. By examining the temporal dependencies between microstate sequences we can investigate how the transitions between functional brain networks are altered in disease. Temporal dependencies are modulated in mood or mental disorders, including FTD (Al Zoubi et al., 2019; Lehmann et al., 2005; Nishida et al., 2013). In Alzheimer disease, in particular, transition patterns appear random while in healthy controls transitions between specific classes are preferred (Nishida et al., 2013).

These findings suggest that EEG microstates have strong potential as a tool for detecting and measuring neural abnormalities in individuals with ALS, particularly as a task‐free assessment of cognitive and behavioural function. Microstate computation exploits the activity that pertains to specific brain regions (by clustering EEG topographies) and therefore microstate classes are hypothesised to reflect specific functional networks, as evidenced by studies examining the relationship between resting‐state networks and microstates. By quantifying microstate properties, we gain the ability to investigate neural network activity.

The purpose of this study was to test whether microstate properties can differentiate ALS and HC groups, in standard characteristics (e.g., frequency of occurrence, duration) and temporal dependencies (e.g., transition probabilities and entropy in microstate sequences). This study also examined whether patients exhibit changes in microstate properties over time and whether microstate properties correlate with clinical presentation. To preface our results, RS EEG microstate analysis suggests that ALS affects both sensory and ‘higher‐order’ networks, resulting in reduced dynamicity in brain state transitions. Microstate properties may be a useful ALS prognostic marker for cognitive decline and disease outcome.

## 2 METHODS

### 2.1 Experiment

#### 2.1.1 Participants

Individuals with ALS and ALS–frontotemporal dementia (ALS‐FTD) diagnoses were recruited from the Irish National ALS Clinic in Beaumont Hospital, Dublin, Ireland. ALS diagnoses were based on the revised El Escorial criteria (Ludolph et al., 2015) and the Strong criteria (Strong et al., 2017). Individuals diagnosed with primary lateral sclerosis, progressive muscular atrophy, flail arm/leg syndromes, other medical morbidities, neurological or neuropsychiatric symptomatology were excluded. Age‐matched healthy controls (HC), with neither diagnosed neurological nor neuropsychiatric conditions, were additionally recruited from an existing volunteer database (Burke et al., 2017). EEG data recorded from 129 individuals with ALS (*m*: 77%; mean age: 60.89 ± 11.4) and 78 age‐matched healthy controls (*m*: 36%; mean age: 60 ± 12) were analysed. Four follow‐up sessions were conducted for patients, ~5.4 ± 2.1 months apart. Patients attended an average of 2 ± 1.2 recording sessions. Detailed information about the demographic of the dataset can be found in Note 1 in Data S1.

#### 2.1.2 Clinical assessments

Individuals with ALS underwent cross‐sectional and longitudinal clinical assessments including the revised ALS functional rating scale (ALSFRS‐R) (Cedarbaum et al., 1999) (*N* = 162), King's stagings (*N* = 161, direct assessment; *N* = 170, with extrapolation from ALSFRS‐R scores; Balendra et al., 2014), and ALS‐specific behavioural and cognitive measurements (*N* = 153) (Traynor et al., 2003). Functional clinical evaluation data were retrieved from the Irish Motor Neuron Disease Registry for the ALS cohort (O'Toole et al., 2008; Rooney et al., 2013; Ryan et al., 2018; Traynor et al., 2003). Edinburgh cognitive and behavioural ALS Screen (ECAS) (Abrahams et al., 2014) and Beaumont behavioural inventory (BBI) (Elamin et al., 2017) scores were collected as part of parallel ongoing research projects in the Academic Unit of Neurology (Costello et al., 2020, 2021) (please see Note 2 in Data S1).

#### 2.1.3 EEG acquisition

Resting‐state EEG recordings were conducted at the Clinical Research Facility in St James's Hospital, Dublin. The EEG recordings occurred in a dedicated recording room, shielded by a Faraday cage to protect from external electric fields. Electrode offsets were kept between ±25 mV. Participants were asked to rest with their eyes open while comfortably seated. A letter X (6 × 8 cm^2^, printed black on white) provided a gaze target. EEG signals were recorded at 512 Hz on a 128 channels BioSemi ActiveTwo system (Amsterdam, Netherlands) (Honsbeek et al., 1998), for three blocks of 2 min. The subject's wakefulness and well‐being were monitored between each recording during a quick visit by the experimenter.

#### 2.1.4 EEG pre‐processing

Pre‐processing was performed using MATLAB R2019b software (The MathWorks, 2019). The EyeBallGUI toolbox (Mohr et al., 2017) was used for visual screening and quality inspection of recordings. The Fieldtrip Toolbox was used for the pre‐processing steps described below (version 20190905) (Oostenveld et al., 2011), and the Microstate EEGlab toolbox (Poulsen et al., 2018) was used to compute the microstates. The pre‐processing steps were implemented based on pipelines previously described in publications by our team (Dukic et al., 2019, 2021; Nasseroleslami et al., 2019). Bad epochs were rejected based on an evaluation of the amplitude, the mean shift, the variance and the band‐variance of spectral power against a 3.5 *Z*‐score threshold (Dukic et al., 2017). The EEG signals were downsampled from 512 to 256 Hz. After resampling, a band‐pass filter (one‐pass zero‐phase FIR: 1–97 Hz) and a notch filter (dual‐pass third‐order Butterworth: 50 Hz, stopband: 1 Hz) were applied.

After baseline correction, noisy channels were removed using an algorithm based on both the PREPpipeline (2015) and the work of Kohe (2010) (Bigdely‐Shamlo et al., 2015; Kohe, 2010). Channels that were removed were interpolated from neighbouring electrodes. Recording sessions with more than 11 channels removed were excluded from the study as they were deemed unreliable. The average number of channels removed was 2.6 ± 6.6 for controls and 3.9 ± 8.6 for patients. A common average reference was applied to the remaining channels.

#### 2.1.5 Computation of the EEG microstates

**FIGURE 1:**
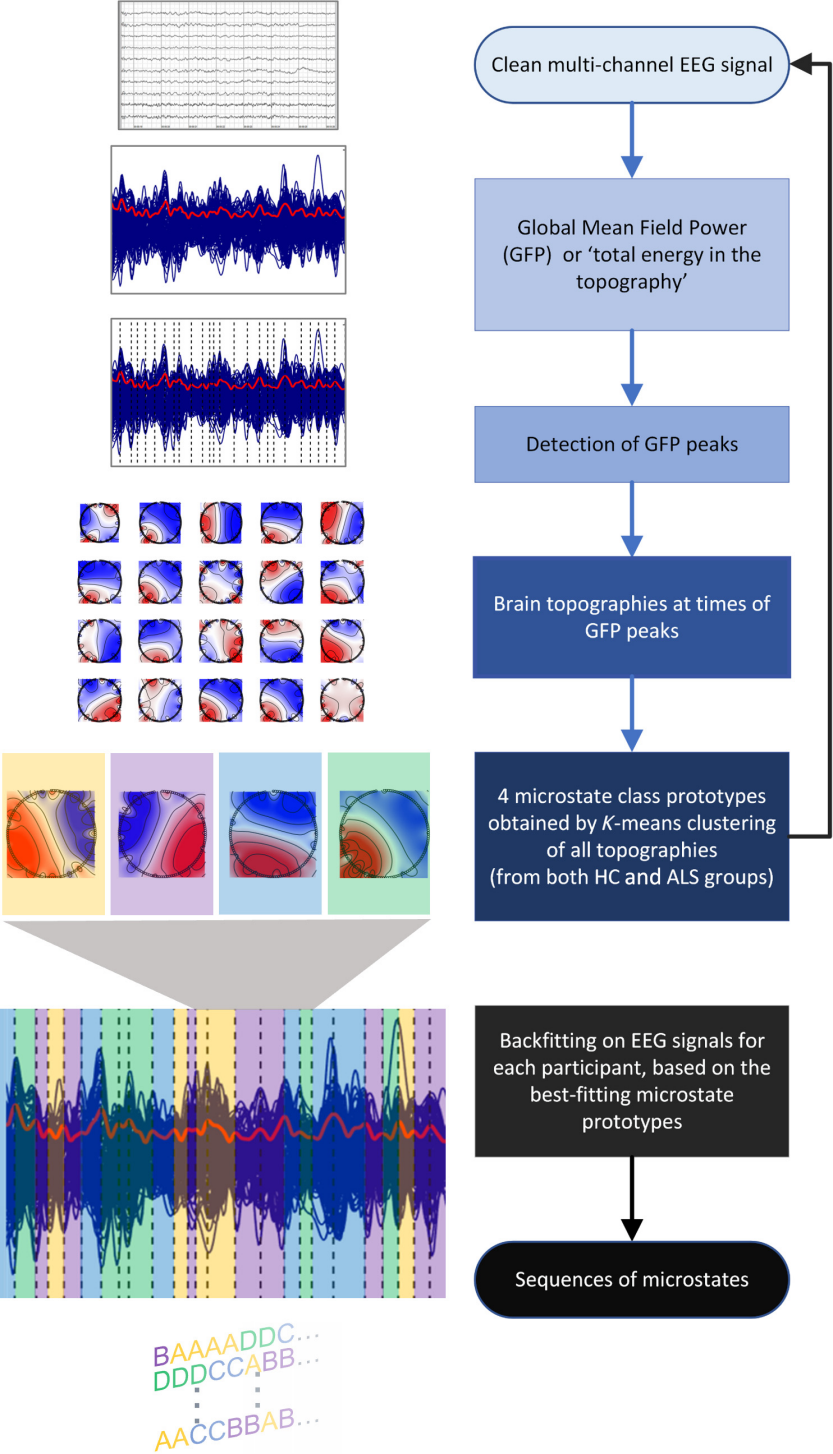
Microstate analysis pipeline. Description of the method used to compute microstates from EEG data. First, a low‐pass filter was applied, and the global mean‐field amplitude (GFP) was calculated for each participant. Next, for each individual, EEG maps were extracted from the signals at 1000 randomly chosen local maxima of the GFP curve and a modified K‐means clustering algorithm was used to cluster the maps of the combined ALS and HC group into microstate classes. Finally, the microstate prototypes were back‐fitted to the original EEG recordings to derive sequences of microstates for each participant.

To compute microstates, EEG data were low‐pass filtered at 30 Hz (zero‐phase, Finite Impulse Response—‘Brickwall’ filter, applied in dual pass form), as commonly recommended in microstate studies (Michel & Koenig, 2018). The computation steps following data pre‐processing are represented in Figure 1. The global mean‐field power (GFP; representing the spatial standard deviation) was calculated for each participant with a Gaussian weighted moving average as a smoothing method (window of five timepoints or around 10 ms) (Al Zoubi et al., 2019). Next, EEG topographies were extracted from the signals at 1000 randomly chosen instances of local maxima of the GFP curve (12% ∓ 2% of the total number of peaks, calculated using a peak‐finding algorithm). Only 1000, rather than all, peaks of GFP were used for each participant to facilitate computation with a relatively large dataset (Poulsen et al., 2018). These EEG topographies at GFP peaks were used to obtain the optimal signal‐to‐noise ratio, whereby peaks higher than 1.5 SDs from the mean were excluded from the selection. Very high GFP often represents non‐neural activity and therefore needs to be rejected. Peaks with <10 ms delay in between were also excluded (Poulsen et al., 2018), as this minimum peak distance guarantees that all peaks are distinct. The selected EEG topographies were submitted to a modified K‐means clustering algorithm, implemented in the Microstate EEGlab toolbox (Poulsen et al., 2018). The algorithm initially defines K microstate prototypes randomly selected from the EEG data. Each EEG sample is assigned to a cluster by minimising the Euclidean distance between the selected EEG maps and the associated prototype. New cluster prototypes are iteratively defined until convergence or a maximal number of repetitions (50 repetitions in our case) is reached. The algorithm models the signal strength and applies a constraint to only have one microstate active at a time. This differs from the original *K*‐means algorithm by being polarity invariant (assigning opposite maps to the same cluster). The rationale for this approach is that the scalp potentials measured by EEG are generated by fluctuations in the synchronous firing of neurons; therefore an inverse polarity of the scalp potential field may happen while the same neuronal sources generate oscillations in the brain (Brodbeck et al., 2012; Michel & Koenig, 2018).

The *K*‐means algorithm was chosen over the agglomerative hierarchical clustering (AAHC) as it has a shorter computational time and both algorithms have been shown to result in similar microstates (Murray et al., 2008). The optimal number of clusters (or microstate classes) was selected using a *K* = 3 cross‐validation approach on a subset of 3–11 maps. Microstate prototypes were identified from two‐thirds of the concatenated GFP peaks and backfitted to the remaining data points (the remaining third of the GFP peaks was the test set), allowing for evaluation of the prototypes' performance on the test set using measures of fit like global explained variance and cross‐validation criterion (Pascual‐Marqui et al., 1995). The cross‐validation method ensures the stability of the results, i.e. not getting microstate cluster representing noise. To derive sequences of microstates, the grand mean across groups prototypes were then back‐fitted to the original EEG recordings for both the HC and the ALS groups. Each EEG sample was associated with a prototype class using global map dissimilarity. Microstate time courses underwent temporal smoothing (using rejection of small segments) to minimize the influence of fast fluctuations, which may be caused by noise. Short microstate of <23 ms (or 6 timepoints) were modified to the next most probable microstate class (Poulsen et al., 2018). While temporal smoothing is beneficial for reducing noise‐related artefacts, it is not suitable when investigating temporal dependencies within the microstate sequence. For this aspect of the study, temporal smoothing was intentionally omitted to preserve the inherent temporal structure of the microstate sequence, following the recommendation by von Wegner et al. (2017).

#### 2.1.6 EEG microstates analysis

After the microstate sequences were computed, two types of analysis were conducted. First, the standard microstate characteristics were extracted, including the global explained variance, occurrence, duration and transition probabilities. Three categories of statistical analyses were conducted on those properties: (1) pairwise comparisons between HC and ALS groups, (2) longitudinal analysis in individuals with ALS over the progression of the disease and (3) cross‐sectional and longitudinal characteristics of the microstate sequences were analysed with respect to the clinical scores.

Second, the temporal dependencies between microstate classes were examined using Shannon entropy and transition probabilities to quantify the predictability and randomness of the microstate sequence (von Wegner et al., 2017). The sequences of microstates were tested for Markovianity of order 0–2. The time‐lagged mutual information between microstates, as well as the stationarity and symmetry of the transition probability matrices were also assessed. These properties have the advantage of being independent of the method used to compute the microstates (von Wegner et al., 2018).

##### Standard properties of microstates

The global explained variance (GEV) measures how well each microstate class can explain the variance in the EEG signal. Basic temporal parameters were determined, including the average duration (ms) of a microstate class, its frequency of occurrence (s^−1^), and the fraction of time it is active during the recording (i.e., coverage). Transition probabilities were also derived from the sequences of microstates to quantify how often one class precedes another. The probabilities were not adjusted for class occurrences or durations, as we chose to report them both independently (Poulsen et al., 2018). Therefore, any observed effects in transition probabilities result from a combination of systematic transition disparities and potential biases explained by occurrences.

###### Statistical analysis

*Cross‐sectional pairwise comparisons*. Mann–Whitney *U* tests were computed for each microstate parameter (coverage, occurrence, duration and transition probability) to compare the HC and ALS cohorts. A 10% adaptive False Discovery Rate (FDR) correction was used to account for the four microstate classes (or 12 transitions between classes), which was based on the Benjamini and Krieger method (Benjamini et al., 2006) as implemented in the Empirical Bayesian inference (EBI) toolbox (Nasseroleslami, 2018). The effect sizes were derived from the *U*‐statistics using the rank‐biserial correlation coefficient (Cureton, 1956): $r=\frac{2U}{n_{1}∙n_{2}}$, as well as the area under the receiver operating characteristic curve (AUROC) (Hajian‐Tilaki, 2013): $\text{AUROC}=\frac{U}{n_{1}∙n_{2}}.$ A post‐hoc EBI‐based estimation of the statistical power was then calculated (Nasseroleslami, 2018).

*Longitudinal changes*. In the ALS group, mixed‐effects models were used to examine the changes in microstate parameters and clinical scores (from ALSFRS‐R, ECAS and BBI tests) over time as the disease progressed. Mixed‐effects models were implemented with an intercept and a time‐related slope, reflecting the rate of change per month (from 5 to 113 months after onset). Mixed‐effects models of the microstate parameters included microstate classes as a predictor. Subject‐specific random‐effects were included in all models: a random intercept was chosen for the longitudinal model to allow for different baseline values across subjects and a random slope was chosen to allow for different rates of change over time. Age, gender and site of onset as random‐effects did not improve the model fit (likelihood ratio test) and were therefore not included in the final models. Education as a random‐effect was deemed relevant for the ECAS model only. A specific deviation from intercept and slope, representing the level of education, was added (as random‐effect) to the model of cognitive performance. The longitudinal model of cognition also contained an additional fixed‐effect term to account for the three different versions of the ECAS questionnaire. The mixed‐effects parameters were estimated using restricted maximum likelihood. The assumptions of normal distributions, independence, and constant variance of the residuals were checked (using the Kolmogorov–Smirnov test [*q* < 0.05]; Ljung‐Box *Q*‐test [*q* < 0.05]; Engle's ARCH test [*q* < 0.05] or diagnostic plots). A rank‐based inverse normal transformation was applied in cases where the residuals did not follow a normal distribution (Beasley et al., 2009). To evaluate the linearity of the parameters' progressions over time, quadratic polynomial regression models were estimated per subject (when data from at least three recordings were available). The quadratic coefficients did not significantly differ from zero (*q* < 0.05), so only first‐order models were kept for further analyses. All patients were included in the final models, regardless of the number of recording sessions they attended as mixed‐effects models can adjust for missing data. To assess the repeatability of the models, the variances of the linear mixed‐effects models were analysed and decomposed to determine the proportion of variance attributed to various sources, including within‐person and between‐person measures (Rights & Sterba, 2021; Schielzeth & Nakagawa, 2022).

*Correlations with clinical measures*. Spearman rank correlations were computed between the microstate parameters and cross‐sectional physical and cognitive clinical scores in the ALS group (survival, ALSFRS‐R and ECAS scores at the first timepoint). The correlation between the variables that describe the microstate properties and clinical scores over time was also estimated. We evaluated correlations separately for those with cognitive impairment (ALSci; based on ECAS score), behavioural impairment (ALSbi; based on BBI scores) and those without cognitive or behavioural impairment, as people with ALS that have extramotor impairments exhibit different changes in functional connectivity (Temp et al., 2021; van der Burgh et al., 2020). An adaptive FDR correction was applied and the statistical power was estimated using EBI (Nasseroleslami, 2018) to account for the multiple clinical measures.

##### Information–theoretical properties to assess temporal dependencies

**FIGURE 2:**
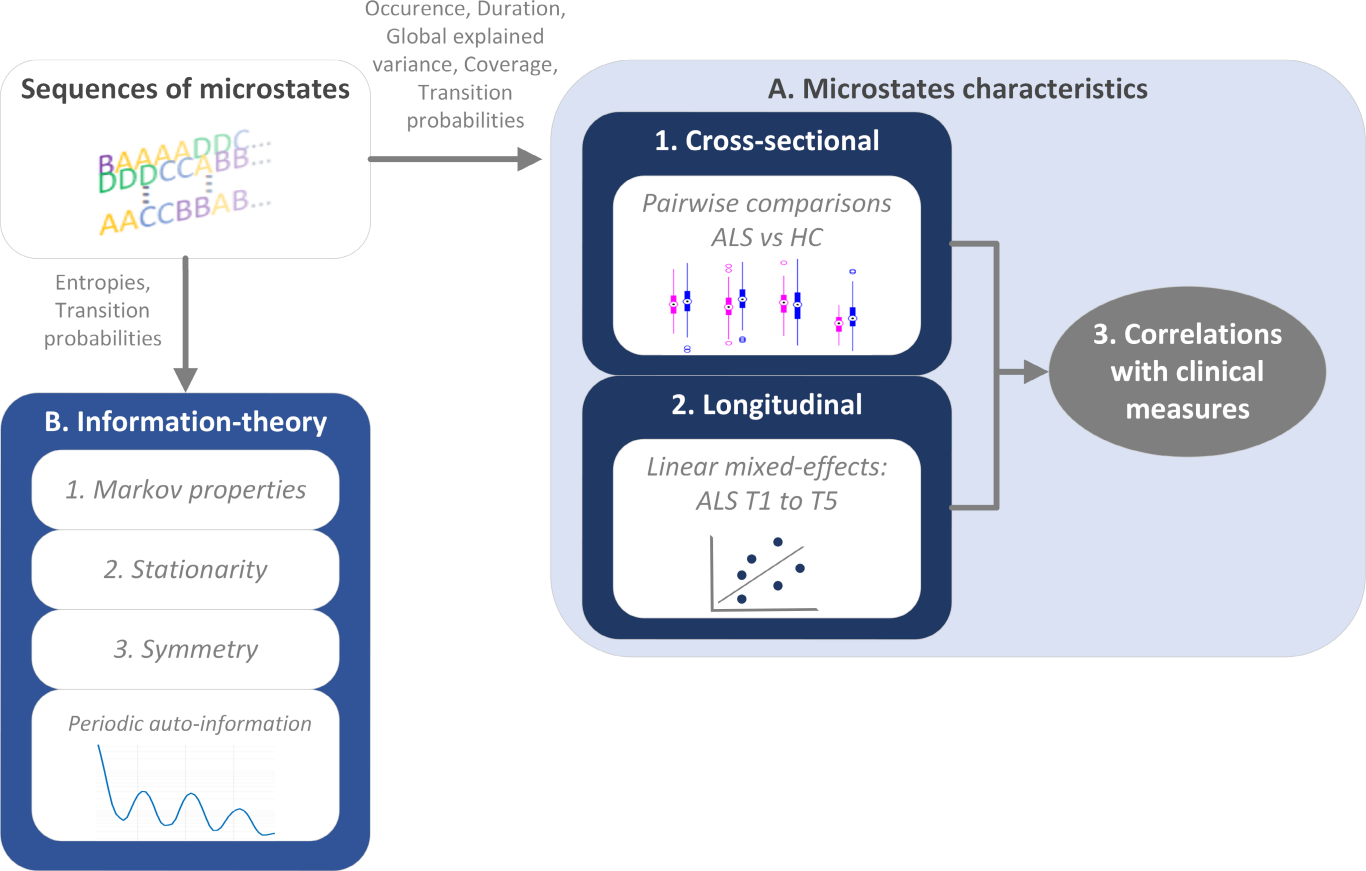
Microstates' stages of analysis. (a) ALS and HC cohorts were compared cross‐sectionally based on the microstate characteristics extracted from the sequences of microstates. The longitudinal changes of the extracted microstate characteristics were additionally examined. Both cross‐sectional and longitudinal characteristics of the microstate sequences were analysed in association with clinical measures. (b) The dynamics hidden inside the sequence of microstates were studied based on information theory.

We performed an information–theoretical analysis of the temporal dependencies between microstate classes using Shannon entropy and by interrogating the transition probabilities (extracting their Markov properties, stationarity and symmetry; Figure 2) (von Wegner et al., 2017, 2018). Studying entropy‐related properties is a way to determine the predictability of the next microstate class. A sequence with only one microstate class appearing (amongst the four classes labelled A, B, C and D) would represent maximum predictability and therefore minimum entropy (e.g., only B). We then derived the auto‐information function (AIF) from the entropy values. AIF measures the time‐lagged mutual information between microstates (it is an approximation of the auto‐correlation function for nonmetric data). The AIF measures the time‐lagged mutual information between microstates with time lag τ, which can be estimated as the difference between the marginal and conditional entropies: $I\left(\tau \right)=H\left(M_{t+\tau }\right)-H\left(M_{t+\tau }\right|M_{t})$. The less ‘uncertainty’ about the time‐lagged microstate $M_{t+\tau }$, when $M_{t}$ is known, the more information is shared between the states and the higher the AIF is. The AIF was evaluated for all microstate classes as well as the contribution to AIF by each microstate class (the time‐lagged mutual‐information for each microstate class separately).

Then we examined the features of the transition probabilities. We first tested for Markovianity order 0–2, to check whether the transition probabilities rely on the current class, the previous class, or two previous classes of the sequence of microstates: with the null hypothesis of no memory effect. The stationarity of the transition probability matrix was then evaluated based on the homogeneity of non‐overlapping blocks of varying lengths. Stationarity means that the frequency of any transition between two classes does not depend on time and would not be significantly different in different blocks (von Wegner et al., 2017, 2018). Finally, the symmetry of the transition matrix was assessed to check whether the probability to transition from a class $M_{i}$ to another class $M_{j}$ was equivalent to the probability of passing from $M_{j}$ to $M_{i}$. Statistical significance for symmetry, stationarity and the Markovianity was estimated using *G*‐tests (i.e., maximum‐likelihood significance tests) and chi‐squared distributions (Al Zoubi et al., 2019; von Wegner et al., 2017, 2018).

## 3 RESULTS

### 3.1 Four microstate prototypes identified in HC and ALS cohorts

**FIGURE 3:**
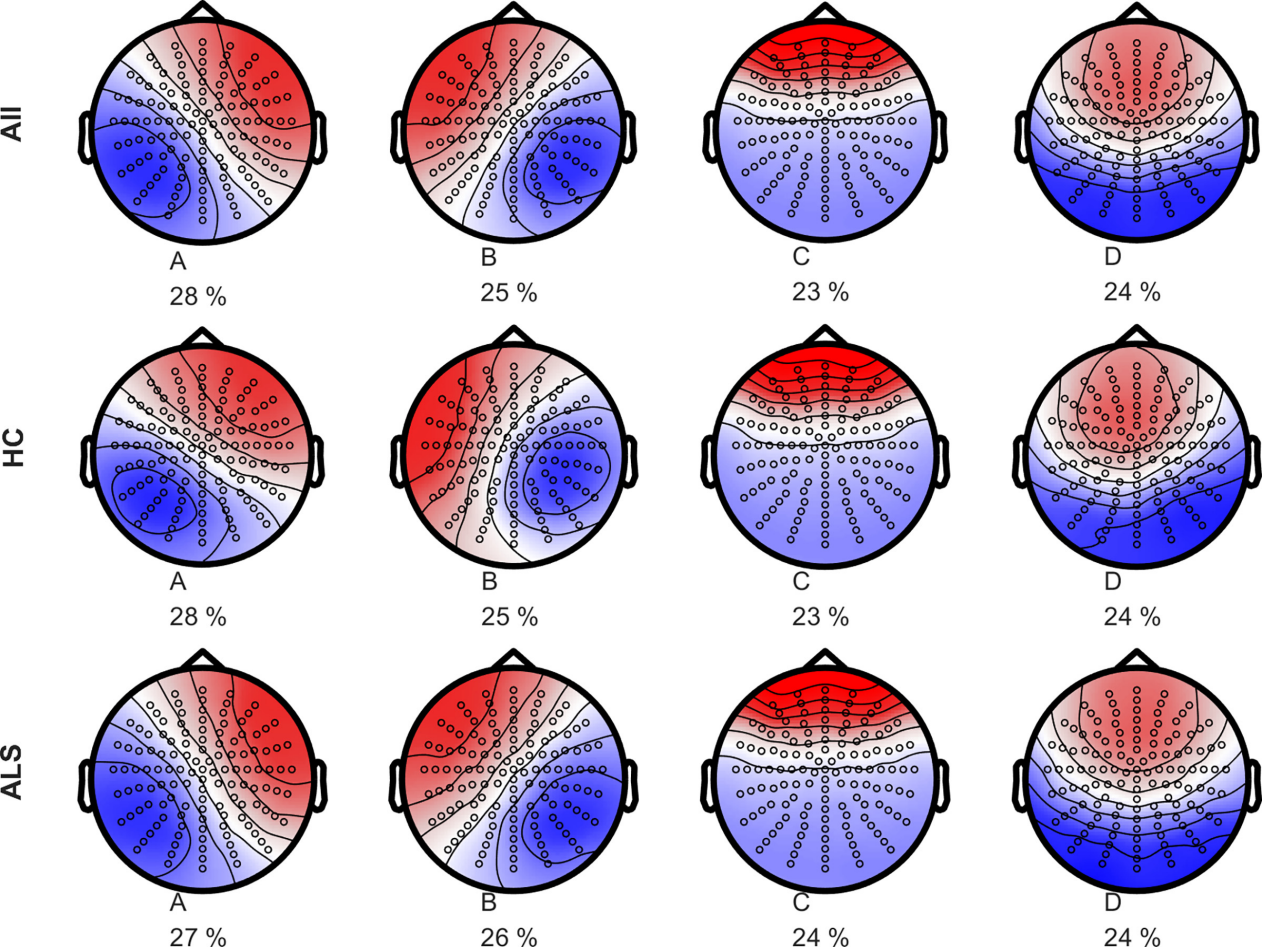
Spatial topographies of the four microstate classes labelled A–D for both the HC and the ALS groups. The polarity is not taken into account. The microstate maps were reordered, according to their topographies, to fit the literature. The contribution of each class to the sequence of microstates is indicated below (in percentage).

The topographies of the microstate prototypes and the optimal number of clusters identified in both HC and ALS groups (Figure 3) were similar to those conventionally reported in the literature (Michel & Koenig, 2018).

The portion of recordings explained by the four microstate prototypes (i.e., explained variance) was 58% for HC and 54% for the ALS group. The four topographies demonstrated spatial correlation between the ALS and the HC groups (Pearson's correlation coefficient: ρ>0.9) and the distributions of the explained variance did not differ between the two groups (2‐sample Kolmogorov–Smirnov test, *p* = .7, respectively).

### 3.2 Modulation of microstate properties by ALS disease

#### 3.2.1 Distinct microstate properties between HC and ALS cohorts

**FIGURE 4:**
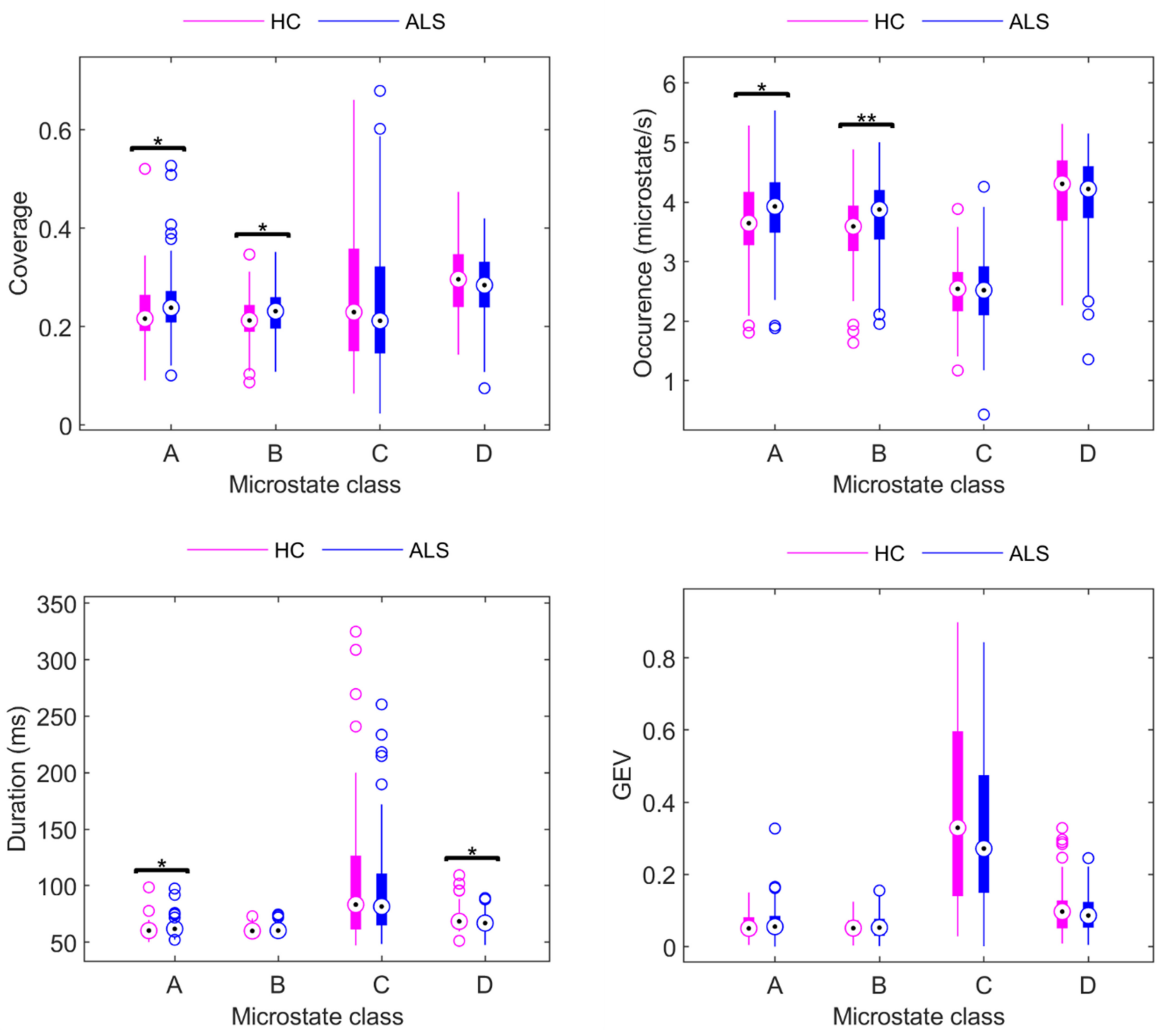
Distributions of specific characteristics for the microstate classes (A–D) for HC and ALS cohorts. Significant differences were observed for the coverage of classes A and B (coverage A: *p* = .02; coverage B: *p* = .03), the occurrence of classes A and B (occurrence A: *p* = .03; occurrence B: *p* = .008), and a significant difference was also observed for the duration of classes A and D microstate (duration A: *p* = .04, duration D: *p* = .02). No significant difference was observed for the GEV of the microstates. All effect sizes were moderate (|*r*| = 0.2). Benjamini and Krieger FDR, *q* < 0.1, was applied. *p ≤ .05; **p ≤ .01.

There were no differences in the GEV distributions of the microstate classes between ALS (measured at the first timepoint) and control groups after FDR correction (*q* < 0.1). Microstate class B, in particular, seems to be most affected by ALS. The occurrences of both microstate classes A and B were higher in the ALS group (Figure 4, occurrence A: *p* = .03, *r* = −.2, 1 − *β* = 0.50, AUC = 0.59; occurrence B: *p* = .008, *r* = −.2, 1 − *β* = 0.65, AUC = 0.60). The coverages of classes A and B were also significantly higher in the ALS group (Figure 4, coverage A: *p* = .02, *r* = −.2, 1 − *β* = 0.53, AUC = 0.59, coverage B: *p* = .03, *r* = −.2, 1 − *β* = 0.48, AUC = 0.59). There was an imbalance between microstate classes – the duration of microstate A was significantly higher in the ALS cohort, whereas the duration of class D was significantly lower (Figure 4, duration A: *p* = .04, *r* = −0.2, 1 − *β* = 0.41, AUC = 0.58; duration D: *p* = .02, *r* = .2, 1 − *β* = 0.48, AUC = 0.60).

**FIGURE 5:**
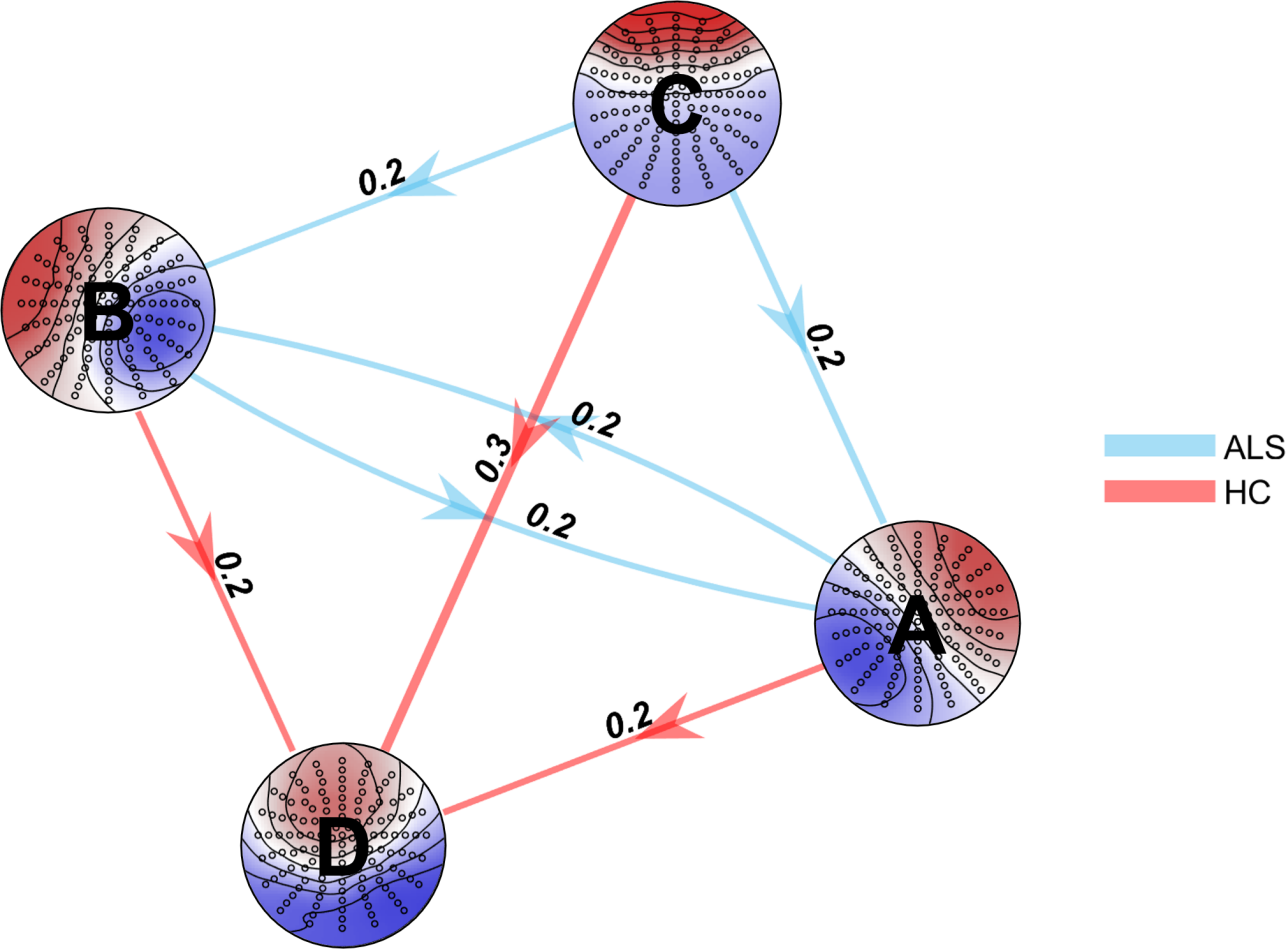
Significant differences in the transition probabilities for the microstates classes (A–D) between HC and ALS cohorts (7 out of 12 transitions). The blue arrows represent higher transition probabilities in ALS, while the red arrows represent higher transitions in controls. Effect size |*r*| are represented above each arrow and the thickness of the arrows is equal to 10 · |*r*|. Benjamini and Krieger FDR, *q* < 0.1. Larger effect (*r* = .3) was observed for the transition from C to D.

The transition probabilities were significantly different between groups for 7 out of 12 transitions (Figure 5). The largest difference between HC and ALS groups was observed for the transition of microstate C to microstate D (p=.004,r=.3,1−β=0.74,AUC=0.63). The transition C → D was more frequent in healthy controls.

#### 3.2.2 Longitudinal changes of microstate properties in ALS

**FIGURE 6:**
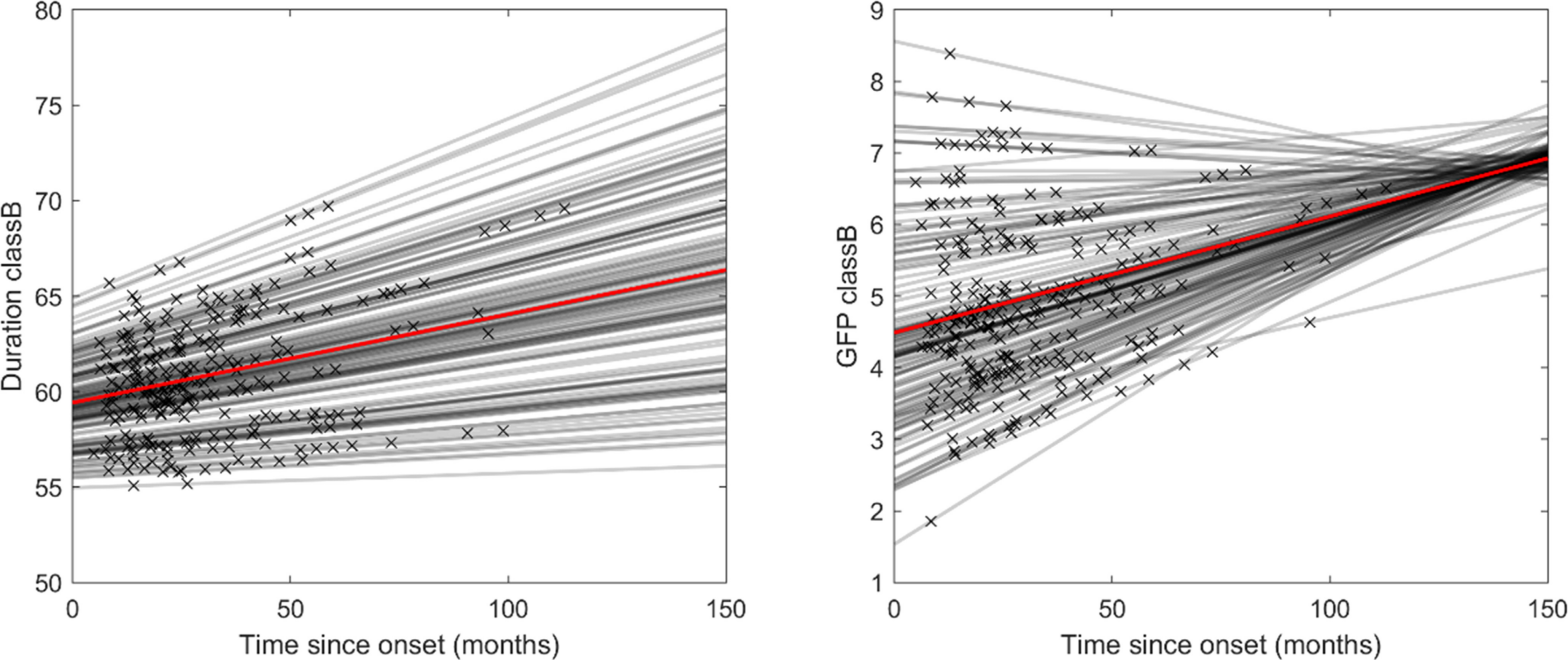
Directions of the significant (*q* < 0.05) longitudinal microstate changes per microstate class. Grey lines represent linear models of microstate property change per participant (based on random‐effects), while red lines represent the overall linear changes (based on fixed‐effects). Crosses represent recording times for each participant. Microstate duration is expressed in ms. Global field power (GFP) is expressed in μV.

The longitudinal analysis of the microstate properties in the ALS group revealed a significant decrease in class B duration (5% increase) and GFP over time (2% increase) (Figure 6). The results emphasized the importance of taking into account different baseline values (using a random intercept model) between individuals and different rates of change over time (using a random slope model). This approach allows to effectively discern the sources of variability. In the longitudinal model of class B duration, the random slope variation accounts for ~1% of the total variance. In addition, roughly 60% of the outcome variance is attributable to person‐specific differences at baseline. Similarly, for class B GFP, 4% of the total variance is attributed to random‐time effects, while 70% is attributed to intercept variation. A summary of the linear mixed‐effects models can be found in Table 1.

**TABLE 1:** Model parameter estimates from longitudinal analyses of the microstates' properties.

	Class B	
	Duration	GFP
Log‐likelihood	−670	−370
*Fixed‐effects*		
Intercept	59 (0.6) ms***	4 (0.2) μV***
Time	**0.05 (0.02) ms/month** *	**0.02 (0.005)** μV**/month** ***
*Random‐effects*		
Intercept variance	3 ms^2^	2 μV^2^
Time variance	0.02 (ms/month)^2^	0.01 (μV/month)^2^
Residual	2 ms	0.6 μV

*Note*: Fixed and random‐effects of the models describing microstate occurrence, duration, coverage, global explained variance (GEV) and global field power (GFP) progressions over the time of the disease were computed. Only models with significant time effects (FDR correction, *q* = 0.05) are shown. Significant time effects are shown in bold and shading. Standard errors were added in parenthesis for fixed‐effects. The analysis included 129 patients and 1020 observations.

*p < .05; **p < .01; ***p < .001.

Gender, age or medication did not have a significant effect on the observed cross‐sectional differences in microstate properties between ALS and HC groups or longitudinal effects in the ALS group (Note 3 in Data S1).

### 3.3 Longitudinal changes of clinical measures in ALS

The clinical scores were also modelled using a linear mixed‐effects model to investigate individual differences in progression (Note 2 in Data S1). As expected, significant time effects were observed for each ALSFRS‐R subscore (*p* < .001) (bulbar, lower limbs, upper limbs, respiratory), with a 0.1 to 0.2 points decline per month. The ECAS Total scores also significantly increased over time (*p* = .02, 0.2 points increase per month) but no increase was observed in the BBI scores (*p* = .05).

#### 3.3.1 Changes in microstate properties are associated with cognitive decline and prognosis

**FIGURE 7:**
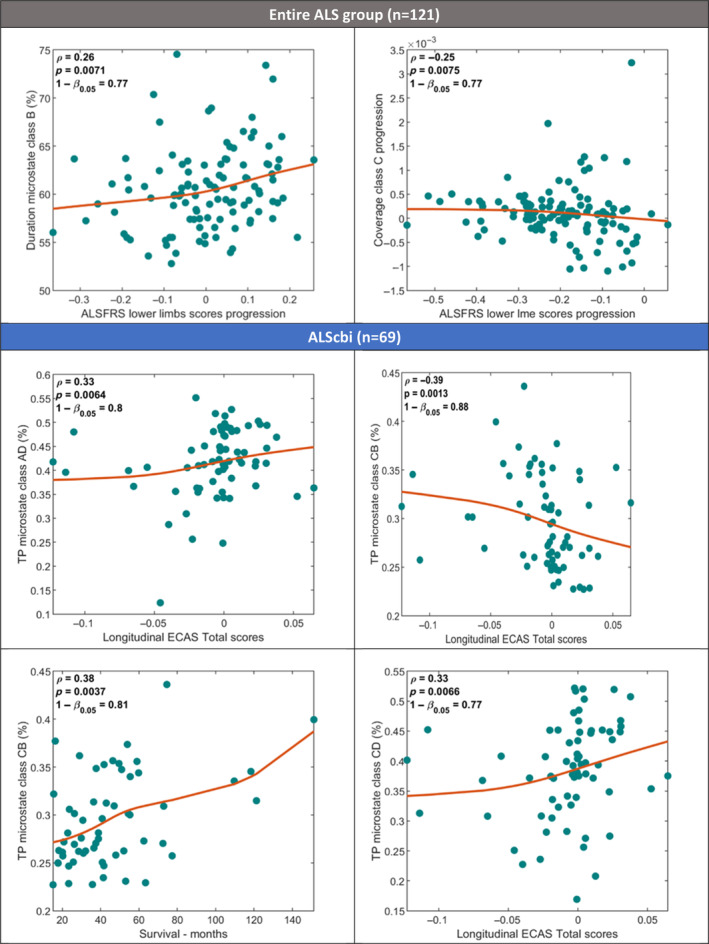
Significant Spearman correlations between clinical scores progressions and properties of microstates classes for ALS cohort and subgroup of patients with distinct cognitive profiles (ALScbi). Solid lines represent monotonically increasing/decreasing spline interpolations used for fitting monotone curves to the data. FDR correction at 0.05. TP, transition probability.

We found that microstates episodes are not only affected by the disease but their characteristics are also associated with the level of cognitive decline. People with ALS who had shorter durations of microstate class B tended to have faster lower motor declines. Individuals with a faster decrease in microstate C coverage had a slower decline in gross motor skills (Figure 7).

Cognitively and behaviourally impaired participants (ALScbi, *n* = 69) with lower transition probabilities in microstate A to D and C to D showed a slower increase in ECAS total scores. ECAS scores generally increase over time due to non‐random dropout or practice effect. At subject level, if these participants showed less of a practice effect it can be interpreted as a sign of cognitive decline. A lower transition probability between microstates C and B was also associated with shorter survival and faster increase in ECAS total scores (Figure 7).

### 3.4 Influence of ALS on temporal dependencies in microstate sequences

#### 3.4.1 Memory effects in the sequences of microstates

For both HC and ALS groups, there were no long‐range memory effects in the microstate sequences, as typically observed (Al Zoubi et al., 2019; von Wegner et al., 2017, 2018). This can be seen in the decay of the periodic peaks of the AIF for time lags larger than 1s (Note 4 in Data S1). The AIF inspection showed that the temporal predictive information in previous time points dependence is less than 1‰ of that in the current time point (>1 s lag).

The Markovianity tests were not significant for any order between zero and two, showing no Markov property (or ‘memoryless’ property, meaning the past is not important as long as the present is known) in the microstate sequences for ALS or HC groups (order 0: p~0; order 1: $p<3.6\times 10^{-72}$; order 2: $p<1.6\times 10^{-26}$). Information from the current microstate is not enough to define the transition probability to the next microstate (Markov order 0). Information from the current and previous microstates is not enough to define the transition probability (Markov order 1). Information from the current and two previous microstates is still not enough to define the transition probability (Markov order 2). The rejection of the null hypotheses in the *G*‐tests for low‐order Markov property reveals memory effects stored at least two microstates in the past.

#### 3.4.2 Reduced dynamicity of microstate transitions in late‐stage ALS

**FIGURE 8:**
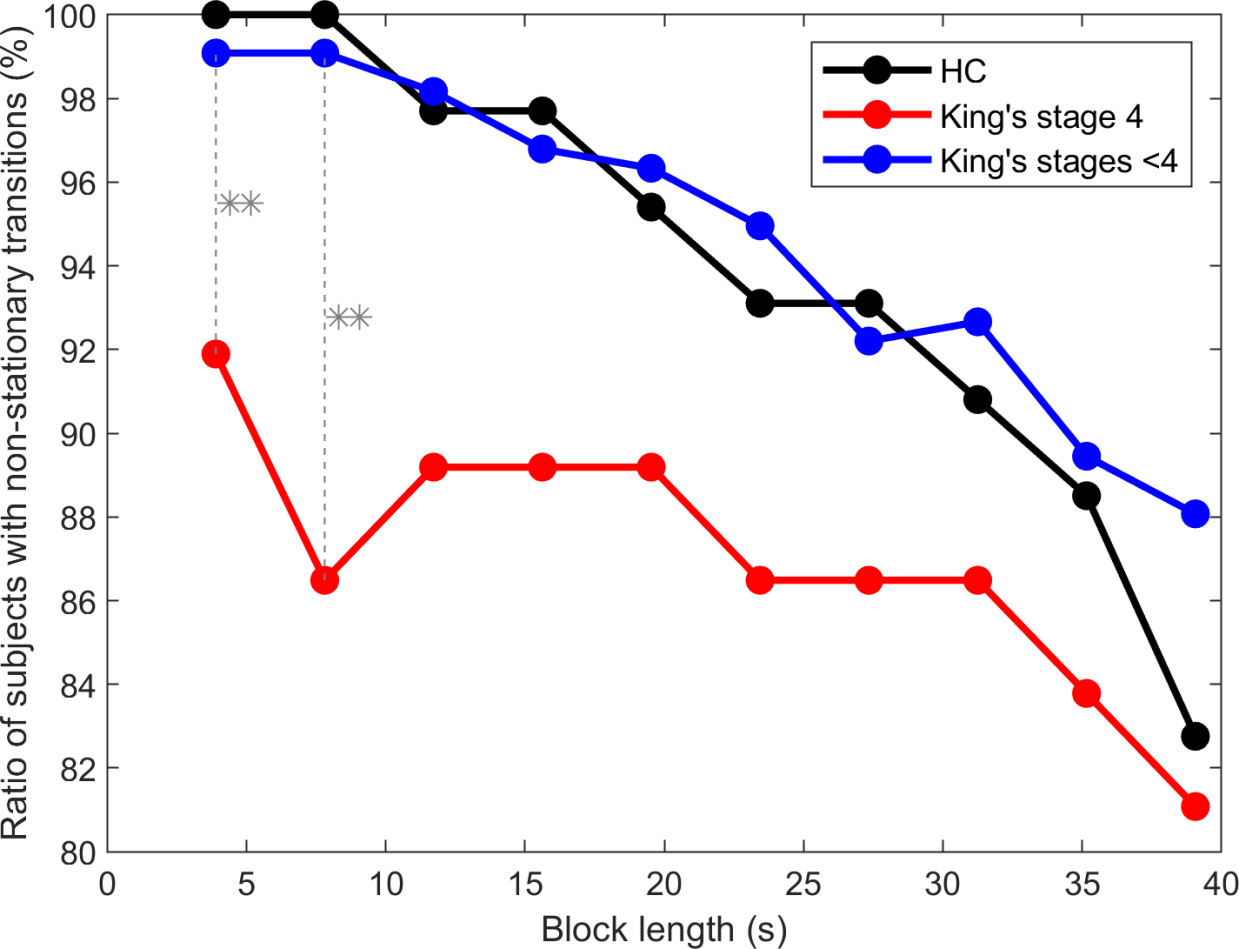
Ratio of subjects (HC, ALS patients in King's stage 4 and lower) with significantly (*p* < .01) non‐stationary microstates transition matrices for different window lengths. For a ~4 s window, significantly more people with King's stage 4 disease (8%) have stationary transition matrices than patients from earlier stages (1%) (Mann–Whitney *U* test, *p* = .0047). Benjamini and Krieger FDR, *q* < 0.05, was applied. *p ≤ .05; **p ≤ .01.

In controls and individuals with early‐stage ALS, the percentage of people with predominantly non‐stationary transition matrices decreased at a similar rate as the block length was increased (where block length is the time window over which the transition probabilities were studied) (Figure 8). Participants in the late stage of ALS (King's stage 4) were more likely to have stationary transition matrices. The frequency of a transition between two classes is staying the same in different blocks, thus becoming independent of time. In ~4 s blocks, significantly more individuals with late‐stage ALS (8%) have stationary transition matrices than individuals from earlier stages (1%) (Mann–Whitney *U* test, *p* = .0032, FDR at 0.05). Higher stability in the transitions between microstate classes has been interpreted as a reduction in the dynamicity of neuronal connectivity (Al Zoubi et al., 2019; von Wegner et al., 2017). No significant difference was observed between the King's stages <4 and the HC group.

For 54% of the HC and 58% of the individuals with ALS, the likelihood of passing from microstate class $M_{i}$ to class $M_{j}$ was not statistically equivalent to the likelihood of transitioning from $M_{j}$ to $M_{i}$. In most participants, the transition matrices were asymmetric. However, only 49% of individuals with late‐stage ALS had asymmetric transition matrices.

## 4 DISCUSSION

The results of this study demonstrate that the properties of EEG microstates can provide insight into ALS prognosis, particularly the degree of cognitive decline over time. The EEG microstates have been examined in a large cohort of people with ALS (*n* = 129) and healthy controls (*n* = 78), enabling a cross‐sectional analysis. This analysis revealed that the standard properties of microstate classes A, B and D differ between ALS and control groups (Figure 4), which may indicate dysfunction in the somatosensory and attention networks. There were also significant differences in microstate transitions between ALS and control groups, Figure 5, suggesting that the normal fluctuations in neural activity are altered in ALS. We also demonstrated that as ALS progresses, the neural dynamics undergo further changes. This is shown by longitudinal changes we observed in the standard properties of microstates (Table 1, Figure 6) and their temporal dependencies (Figure 8). Participants with late‐stage disease showed more symmetry and stationarity in their transition matrices (Figure 8), which could reflect reduced neuronal flexibility (dynamicity in switching between brain microstates).

Finally, the correlations between microstate properties and ALS prognosis revealed that higher duration of class B and faster increase of class C coverage over time are associated with a slower decline in gross motor skills in ALS. For cognitively and behaviourally impaired patients, lower transition probabilities from A to D, C to B and C to D are specifically associated with cognitive decline. This suggests that the microstate parameters have particular potential for development as prognostic biomarkers for ALS.

### 4.1 Changes in microstate properties in ALS

We found that four cluster prototypes (Figure 3) explained ~60% of the variance and exhibited similar topographies in healthy controls and ALS groups (they were also similar to the maps described in the literature, see review; Michel & Koenig, 2018). In studies including more topographies, the four maps initially found in 1999 (Koenig et al., 1999) are usually observed along other topographies, independently of ages, mental states or neurological conditions (Al Zoubi et al., 2019; Custo et al., 2014; Faber et al., 2021; Zanesco et al., 2020). The original A‐D labels were kept based on topographical similarity to the initial maps. Consistent with previous studies (Michel & Koenig, 2018), we observed that four microstate prototypes explained at best the variance of topographical patterns in unrelated data. This cross‐validation check of the optimal number of microstates ensured the microstate prototypes were not representing recording noise (Poulsen et al., 2018).

#### 4.1.1 Distinct microstate properties between HC and ALS cohorts

The statistically significant increase in microstate class A duration and microstate class B coverage in the ALS group, when compared with healthy controls, is similar to what was observed in Parkinson disease (Chu et al., 2020), and in multiple sclerosis studies (Gschwind et al., 2016). The increase in class A and B coverage has also been demonstrated in Huntington's disease (Faber et al., 2021), and an increase in class A occurrence has been documented in both schizophrenia (Lehmann et al., 2005) and spastic diplegia (Gao et al., 2017).

The results of previous fMRI‐EEG studies suggest that class A originates from the bilateral temporal gyri (Britz et al., 2010), occipital and posterior cingulate areas (Pascual‐Marqui et al., 2014) or the sensorimotor cortex (Yuan et al., 2012). Diverse interpretations of microstate class A's functional role have been reported; initially linked with the auditory network (Britz et al., 2010; Custo et al., 2017), a broader involvement including visual processing has been suggested, due to its increased coverage during visualization‐oriented tasks compared with verbalization tasks (Milz et al., 2017). Both of these interpretations would situate the sources of class A microstate within the sensory network, which is known to be affected in ALS. A recent review analysis conducted by Tarailis et al. has further proposed a potential link between microstate class A and varying levels of brain arousal or alertness (Tarailis et al., 2023). Class B is thought to originate in the occipital lobe and is associated with visual function (Britz et al., 2010). Both microstates A and B appear to reflect the activation of sensory networks, as indicated by their modulations in multiple sclerosis (Gschwind et al., 2016) and movement disorders in general (ALS, Huntington, Parkinson and spastic disorder).

Microstate class D occurrence was higher in the ALS cohort than in HC. A high contribution of fronto‐parietal areas and anterior/posterior cingulate cortices (Britz et al., 2010; Pascual‐Marqui et al., 2014) was observed during microstate class D, which altogether suggest an association of microstate D with the attention network. Microstates classes C and D have been associated with ‘high‐order functional networks’ (as opposed to somatosensory or motor networks) (Michel & Koenig, 2018). The balance between such microstate classes was observed to be affected by neuropsychiatric conditions like schizophrenia or FTD (Nishida et al., 2013). While ALS is not primarily classified as a psychiatric disorder, the condition can often present with cognitive and behavioural symptoms.

Taken together the cross‐sectional comparisons of microstate properties between ALS and HC cohorts echo the dual impairment of sensorimotor and cognitive functions in ALS.

#### 4.1.2 Longitudinal changes of microstate properties in ALS

For individuals with ALS, the duration and the GFP of class B significantly increased by 0.05 and 0.02 points per month (Table 1). Neither of those properties was significantly different between the HC group and ALS group at the first recording session. The microstate properties showing significant differences between ALS and HC groups did not reveal any longitudinal change. This finding suggests the presence of important neuronal changes early in the disease, leading to distinct microstate properties in the ALS and HC groups. There may be slower or delayed continuous mechanisms causing changes in other microstate properties. Since early degeneration is usually compensated by remaining neuronal networks in neurodegeneration, such slower mechanisms may be compensatory. In ALS, symptoms only become apparent when a resilience threshold is crossed (Benatar et al., 2022; Keon et al., 2021).

### 4.2 Altered microstate dynamics in ALS

Previous literature has shown that there are differences in microstate transition probabilities in mood or mental disorders (Al Zoubi et al., 2019; Lehmann et al., 2005) and FTD (Nishida et al., 2013), and we hypothesised that the transition probabilities would also be altered in participants with ALS that exhibited cognitive and behavioural symptoms. As expected, we observed significant differences in microstate dynamics between ALS and HC groups in 7 out of 12 of the transition probabilities (*q* < 0.1, FDR correction) (Figure 5). More specifically, we observed that patients switch less frequently from microstate C to microstate D Figure 5.

The results of previous studies on stroke, which reported no significant difference in transition probabilities compared with controls (Hao et al., 2022), suggest that the temporal dynamics of neural networks are not solely due to structural changes.

In this study, we employed the information–theoretical analysis proposed by von Wegner et al. to further investigate the dynamics of EEG microstates (von Wegner et al., 2017). Our findings align with their results indicating that the microstate sequence does not adhere to a low‐order Markov property, suggesting that microstate labelling is influenced by not only the current state or the current and last two states, but also previous states. Furthermore, our analysis of the auto‐information function revealed non‐Markovian behaviour for time lags of up to 2 s, consistent with previous research (Al Zoubi et al., 2019; von Wegner et al., 2017), indicating the presence of extended short‐range memory effects in the microstate sequences.

For the majority of the subjects (HC and ALS cohorts with King's stages <4), the transition matrices were asymmetric. This has been previously interpreted as a sign of ‘non‐equilibrium’ of the neural networks (von Wegner et al., 2017). A lack of symmetry in transition matrices has been interpreted as a positive property, implying the existence of a ‘driving force’ (if there were no ‘driving force’, and the neural networks were at equilibrium, the transition from one state to a second state would be equal to the transition from the second state to the first one). It is not surprising therefore that the late‐stage group (King's stages 4) tended to have more patients with symmetric and stationary transition matrices, Figure 8. The increased number of symmetric and stationary transition matrices observed in late‐stage ALS may correspond to the dysfunction of this ‘driving‐force’. The thalamus, in particular, has been described as a key relay of energy, and could represent a hypothetic ‘driving‐force’ (von Wegner et al., 2017) (thalamic involvement has been demonstrated in motor neuron diseases [Chipika, Christidi, et al., 2020; Chipika, Finegan, et al., 2020; Deymeer et al., 1989]).

The observed change in microstate transitions in late‐stage disease could also be explained by the distress individuals with ALS may experience toward the end of their life. A higher ratio of symmetrical and stationary matrices in individuals with mood and anxiety disorders compared with healthy controls has been similarly shown by (Al Zoubi et al., 2019), which they interpreted as arising from ‘ruminative thoughts’. Increased equilibrium could additionally arise due to a reduction in the flexibility of brain dynamics in ALS. A previous study has shown that the incidence of ‘neuronal avalanches’, a measure of brain dynamics determined by quantifying aperiodic bursts of neuronal activity diffusing across the brain, was reduced in ALS compared with healthy controls cohort and was associated with disease stage (Polverino et al., 2022).

### 4.3 Clinical relevance of EEG microstates

The main finding from the analysis of the correlation between microstate parameters and clinical measures was that lower duration of microstate class B and slower change in coverage of class C were significantly associated with faster functional decline in the lower limbs (Figure 7). These measures, therefore, have potential utility in prognostic prediction of motor function.

We evaluated correlations with clinical scores specifically for subgroups of ALS patients with distinct cognitive profiles as altered microstates characteristics have been specifically associated with impaired cognition and mental health (Al Zoubi et al., 2019; Dierks et al., 1997; Nishida et al., 2013; Tait et al., 2020). In cognitively and behaviourally impaired patients, the lower transition probabilities A to D, and C to D are additionally associated with cognitive decline. This decline is suggested by the gradual improvement in cognitive performance (measured by ECAS Total scores), which is slower when compared with the average practice effect. Additionally, a lower transition rate from C to B was associated with shorter survival (Figure 7).

The transition probability C → B appear to be a key potential biomarker of ALS prognosis. Higher transition probabilities from C to B seem to represent signs of slower decline in ALS. This supports our hypothesis that changes in microstates dynamics could predict the progression of ALS, including cognitive decline.

### 4.4 Limitations and future directions

The EEG microstate analysis is based on a repeatedly observed phenomenon representing ongoing thought processes. However, there remains a lack of understanding of the neural mechanisms leading to the presence of microstates and their transitions. It remains unclear how microstates actually reflect conscious thoughts, despite new insights on microstates in various states of consciousness (e.g., sleep, anaesthesia, wakefulness) (Bréchet & Michel, 2022) and rough estimations of the brain sources each microstate class originate from (Bréchet et al., 2020; Britz et al., 2010; Custo et al., 2017; Milz et al., 2017; Musso et al., 2010; Pascual‐Marqui et al., 2014). The interpretation of microstates' characteristics often relies heavily on estimated brain sources. Previous studies of the brain sources underlying different microstates have reported diverse findings, possibly as a result of differences in methodology and/or lack of temporal independence (difficulty of dissociating microstate sources as microstates are a continuous process). This complicates the interpretation of microstate changes (Britz et al., 2010; Mishra et al., 2020; Yuan et al., 2012). Microstates are fundamentally defined based on sensor space analysis. Therefore, for a precise association with brain sources, other methods can provide more information, such as examining patterns of activation directly in brain networks' functional connectivity. In this study, over‐interpretation was carefully avoided by cross‐examining microstates' hypothetic generators with paradigm‐based studies.

One important consideration is the possible non‐random dropout within the ALS cohort over time, wherein individuals with greater impairments are more likely to be lost to attrition. In the case of longitudinal ECAS scores, the observed increase may not solely be attributed to the practice effect but could also be influenced by artificial inflation of cognitive scores due to the dropout of more impaired participants. However, this potential bias is mitigated when examining correlations between EEG and clinical measures progressions at the subject level, as both are expected to be similarly affected by non‐random dropout.

A limitation of the present study is the heterogeneity of onsets and cognitive/behavioural ALS profiles. In future studies, a more continuous collection of data should help to account for a greater number of clinical profiles and we envisage that a comparison of microstates in different ALS subphenotypes will be possible.

## 5 CONCLUSION

These RS EEG microstate results indicate that ALS impacts both sensory and higher‐order networks. These findings are consistent with the range of motor, respiratory, and cognitive impairments observed in ALS clinical presentations. Temporal dynamics of resting state EEG enable us to further quantify the multidimensional impairments. Importantly, we found reduced dynamicity in brain state transitions, which may occur as a result of declining cognition, repetitive thoughts, anxiety, or neuronal loss. We have shown that changes in microstate properties are associated with cognitive decline and prognosis, making them a promising prognostic marker for ALS.

## AUTHOR CONTRIBUTIONS

Marjorie Metzger, Bahman Nasseroleslami, Orla Hardiman, Niall Pender, Muthuraman Muthuraman, Peter Bede: Conceptualisation. Marjorie Metzger, Stefan Dukic, Roisin McMackin, Eileen Giglia, Matthew Mitchell, Saroj Bista, Emmet Costello, Colm Peelo, Yasmine Tadjine, Vladyslav Sirenko, Serena Plaitano, Amina Coffey, Prabhav Mehra: Investigation (Data acquisition). Marjorie Metzger, Bahman Nasseroleslami: Methodology. Marjorie Metzger: Formal Analysis. Roisin McMackin, Lara McManus, Mark Heverin, Bahman Nasseroleslami, Orla Hardiman: Project Administration. Bahman Nasseroleslami, Orla Hardiman: Resources. Bahman Nasseroleslami, Orla Hardiman, Niall Pender, Muthuraman Muthuraman, Peter Bede: Funding Acquisition. Marjorie Metzger, Stefan Dukic, Bahman Nasseroleslami: Software. Bahman Nasseroleslami, Orla Hardiman: Supervision. Marjorie Metzger: Validation. Marjorie Metzger: Visualisation. Marjorie Metzger: Writing‐original draft. Marjorie Metzger, Lara McManus, Bahman Nasseroleslami, Orla Hardiman: Writing‐review and editing.

## FUNDING INFORMATION

Funding for this study was provided by the Thierry Latran Foundation (Project award to Orla Hardiman), the Health Research Board of Ireland (HRA‐POR‐2013‐246; MRCG‐2018‐02), the Irish/UK Motor Neurone Disease Research Foundation (IceBucket Award; MRCG2018‐02 and McManus/Apr22/888‐791 to Lara McManus and McMackin/Oct20/972‐799 to Roisin McMackin), Irish Research Council (Government of Ireland Postdoctoral Research Fellowship GOIPD/2015/213 to Bahman Nasseroleslami and Government of Ireland Postdoctoral Postgraduate Scholarship GOIPG/2017/1014 to Roisin McMackin) and Science Foundation Ireland (16/ERCD/3854 and Royal Society/SFI URF\R1\221917 to Lara McManus). Peter Bede and the Computational Neuroimaging Group are supported by the Health Research Board of Ireland (Emerging Investigator Award HRB‐EIA‐2017‐019), the Irish Institute of Clinical Neuroscience (IICN) – Novartis Ireland research grant, The Iris O'Brien Foundation and The Perrigo clinician–scientist research fellowship. Muthuraman Muthuraman is supported by the German Collaborative Research (DFG‐CRC‐1193 and CRC‐TR‐128).

## CONFLICT OF INTEREST STATEMENT

No conflict of interest to disclose.

## Supporting information


**Data S1.** Supporting Information.

## Data Availability

The data that support the findings of this study are available from the corresponding author on reasonable request from qualified investigators. Data sharing is subject to the participant's consent and approvals by the Data Protection Officer and the Office of Corporate Partnership and Knowledge Exchange in Trinity College Dublin. The code used to compute the microstates for the analyses described in this article can be found at: https://github.com/atpoulsen/Microstate-EEGlab-toolbox. We additionally adapted the Python code freely available at https://github.com/Frederic-vW/eeg_microstates to MATLAB.
