# Supplementary material for: Functional network dynamics revealed by EEG microstates reflect cognitive decline in amyotrophic lateral sclerosis
Source: Hum Brain Mapp. 2023 Dec 13;45(1):e26536. doi: 10.1002/hbm.26536 (PMC10789208; doi:10.1002/hbm.26536)
Supplement: Supplementary file 1 — Data S1. Supporting Information. [file HBM-45-e26536-s001.docx]

Supplementary materials

# Supplementary note 1: Details of subjects demography

Table 1: Demographic profile for controls and patients. Up to five recording sessions were scheduled for the patients with in-between time delays representing delays between each session. The table details the gender proportions, the average ages at recording and, when applicable, disease durations, delays between sessions, site of onset and the number of patients with FTD comorbidity. Numbers show mean ± standard deviation.

| Groups | N | Male (%) | Age (years) | Disease duration (months) | Follow-up intervals (months) | ALSFRS-R scores | Site of onset (N) | | | ALS-FTD diagnosis (N) |
| --- | --- | --- | --- | --- | --- | --- | --- | --- | --- | --- |
|  |  |  |  |  |  |  | ***Bulbar*** | ***Spinal*** | ***Thoracic*** |  |
| Controls | 78 | 36 | 60 ∓ 12 | / | / | / | / | / | / | / |
| Patients T1 | 121 | 75 | 62 ∓ 11 | 25 ∓ 18 | / | 36 ∓ 7 | 22 | 86 | 5 | 5 |
| Patients T2 | 60 | 77 | 60 ∓ 11 | 32 ∓ 19 | 4.9 ∓ 1.2 | 35 ∓ 8 | 14 | 43 | 2 | 2 |
| Patients T3 | 45 | 80 | 61 ∓ 12 | 37 ∓ 19 | 5.3 ∓ 2.8 | 33 ∓ 9 | 8 | 34 | 1 | 2 |
| Patients T4 | 22 | 86 | 61 ∓ 11 | 42 ∓ 24 | 4.9 ∓ 1.1 | 33 ∓ 7 | 2 | 19 | 1 | 1 |
| Patients T5 | 7 | 57 | 57 ∓ 13 | 52 ∓ 31 | 6.5 ∓ 2.4 | 33 ∓ 6 | 0 | 6 | 1 | 0 |

# Supplementary note 2: Details of clinical assessments

## ALSFRS-R

The revised ALS-functional rating scale (ALSFRS-R) (Cedarbaum et al., 1999) evaluates a patient’s physical function through 12 measures rated from 0 (no ability) to 4 (normal). Measures include the capacity to swallow, use utensils, climb stairs or breathe, and were grouped to be anatomically relevant. We defined subscales of the total ALSFRS-R rate (over 48) as follows: bulbar (scores 1-3), upper limbs (scores 4-6), lower limbs (scores 7-9) and respiratory (scores 10-12). The patients' ALSFRS-R scores were recorded on average 7.7 +/- 5.1 times, between 3.7 and 145 months after onset. The symptoms were evaluated at intervals of approximately 3.3 +/- 3.9 months.

## King’s staging

The King’s clinical staging system (Roche et al., 2012) similarly estimates the anatomical progression of the disease. Stages are defined based on the number of affected regions, from 1 (early disease) to 5 (death).

## Edinburgh cognitive and behavioural ALS screen (ECAS)

The Edinburgh Cognitive and Behavioural ALS Screen (ECAS) (Abrahams et al., 2014) is widely used to detect cognitive and behavioural changes, specifically in ALS patients. The ECAS scores were obtained up to three times, between 3.7 and 100 months after onset. The ECAS Total score, ranged between 46 and 135. Participants with ALS cognitive status were assessed using abnormality cut-off scores, defined based on age and education for the Irish population (Pinto-Grau et al., 2017). Three alternate versions of the cognitive screening tool (A, B and C) allowed serial administration, at 4-month intervals (minimum) whilst minimising practice effects (Costello et al., 2020; Crockford et al., 2018).

## Beaumont behavioural inventory (BBI)

The BBI questionnaire was specifically designed to evaluate behavioural changes in ALS while taking into account (i.e. controlling for) the influence of motor impairment (Elamin et al., 2017). Evaluations were made up to three times per patient, between 2.8 and 100 months after onset, with a score range of 0 to 73. Scores above 6 correspond to mild impairment while scores above 22 show severe behavioural changes.

## Longitudinal analysis of the clinical assessments

Table 2: Model parameter estimates from longitudinal analyses of clinical scores. Fixed and random-effects of the models describing clinical scores (ALSFRS, ECAS, BBI) progressions over the time of the disease. Standard errors were added in parenthesis. *p < 0.05; ***p < 0.001.

|  | | bulbar  ALSFRS | lower limbs  ALSFRS | upper limbs  ALSFRS | respiratory  ALSFRS | total ECAS | BBI |
| --- | --- | --- | --- | --- | --- | --- | --- |
| log-likelihood | | -1883 | -2125 | -2107 | -2259 | -1200 | -1053 |
| *Fixed-effects* | | | | | | | |
| Intercept | | 12 (0.2) *** | 12 (0.3) *** | 12 (0.3) *** | 12 (0.3) *** | 102 (3) *** | 11 (1.5) *** |
| Version B | | - | - | - | - | 1 (1) | - |
| Version C | | - | - | - | - | 0.2 (1) | - |
| Time (per months) | | **-0.1 (0.01) ***** | **-0.2 (0.01) ***** | **-0.2 (0.01) ***** | **-0.1 (0.01) ***** | **0.2 (0.06) *** | **-**0.06 (0.05) |
| *Random-effects* | | | | | | | |
| Subject | *Intercept variance* | 2 | 4 | 3 | 3 | 13 | 11 |
|  | *Time variance  (per months^2^)* | 0.1 | 0.1 | 0.2 | 0.1 | 0.1 | 0.07 |
| Education | *Intercept variance* | - | - | - | - | 8 | - |
|  | *Time variance  (per months^2^)* |  |  |  |  | 0.02 |  |
| Residual | | 0.9 | 2 | 1 | 1 | 5 | 8 |

# Supplementary note 3: Gender, age and medication

To evaluate the effect of age, gender or medication on the observed EEG microstate properties, additional statistical tests were performed (Fisher’s test, Kruskal-Wallis analysis of variance and linear regression). No significant differences in the distributions of age or medication were observed between HC and ALS patients or between ALS patient subgroups. No significant differences in gender distribution were observed between ALS patient subgroups but a significant difference was observed between HC and patients.

We applied linear modelling to verify if the pairwise differences between patients and controls were driven by the significant differences in gender. For each microstate class and parameter, we assessed the differences between HC and ALS patients (at T1), while controlling for gender. In Wilkinson notation, the model would be described as: Parameter ~ Group + Gender. The rank-based inverse normal transformation was applied to ensure the normality of the observations. The previously observed group effects, by pairwise comparisons, were again significant while controlling for gender (FDR, q=0.05). No significant gender effect was observed. The observed differences between ALS patients and HC were not induced by gender, age or medication.

| 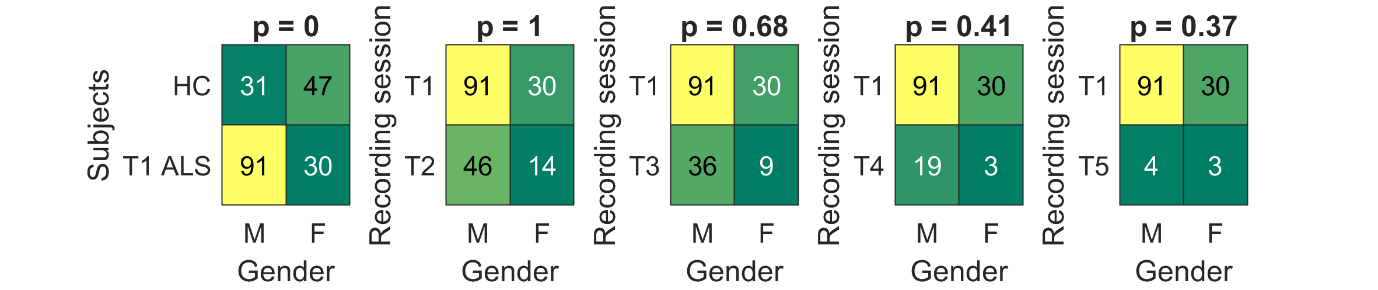   1. *Gender distributions in controls and in patients different recording sessions (T1-T5). Fisher’s exact test (*$\alpha$*=0.05, two-tailed) did not reveal any non-random association between gender and recording sessions for patients follow-ups.* |
| --- |
| 1. 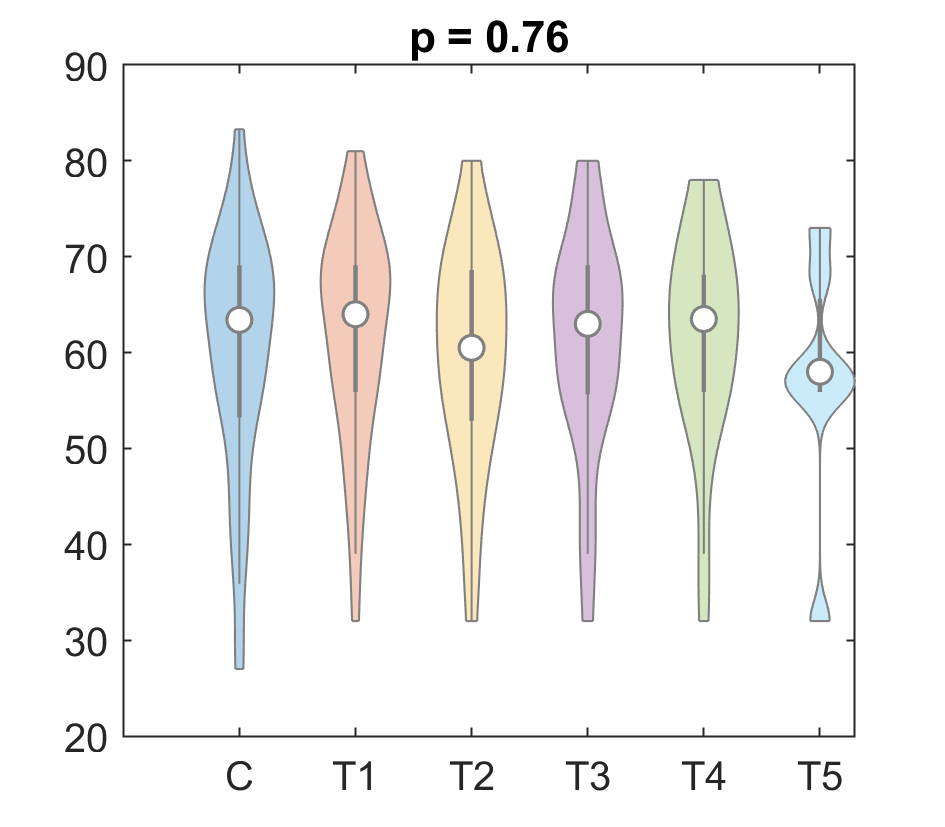*Age distributions in the HC group and ALS group’s different recording sessions (T1-T5). Kruskal-Wallis one-way analysis of variance revealed no statistical difference in age distribution between recording sessions.* |
| 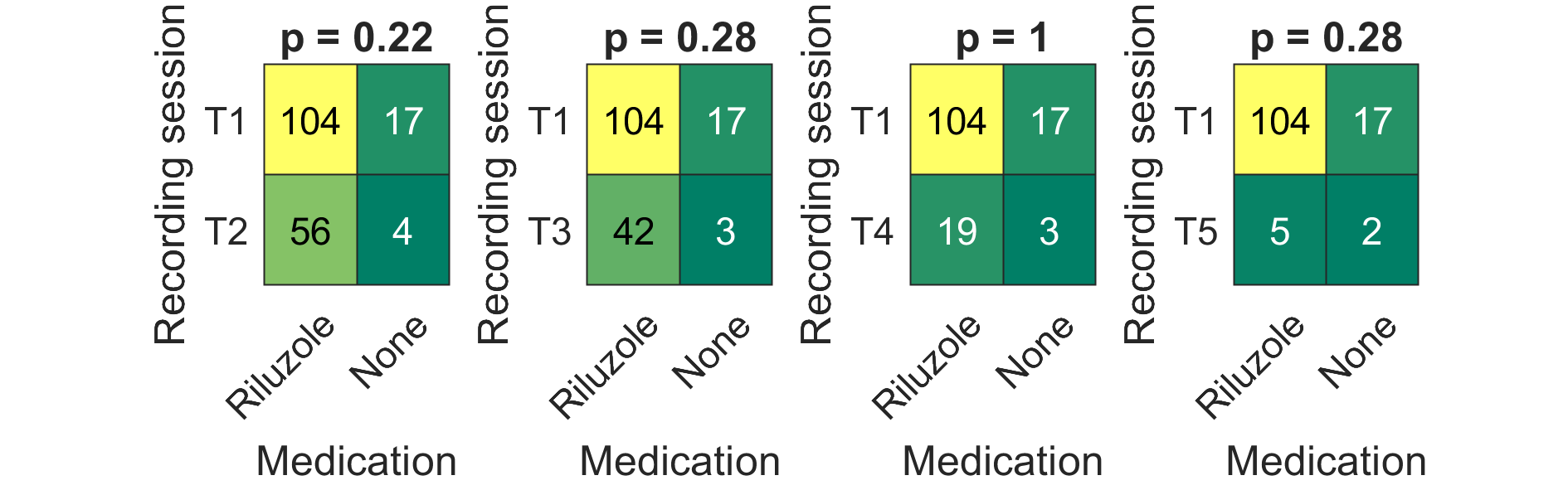*(c) Medication distributions in the ALS group’s different recording sessions (T1-T5). Fisher’s exact test (*$\alpha$*=0.05, two-tailed) did not reveal any non-random association between medication and recording sessions.* |

# Supplementary note 4: Auto-information function

The existence of a ‘memory effect’ in the microstate sequence was estimated based on the AIF, by evaluating how much knowing the label at time $t$ reduces uncertainty at time $t+\tau$. The subject-specific AIF revealed an oscillatory decay in function of the time lags (Figure 1, Control C47), with an average period of 35 +/- 5.5 ms for HC and 33 +/- 5.2 ms for ALS patients. For both HC and ALS groups, we observed a decay of the periodic peaks for time lags larger than $1s$ (Figure 2). In line with previous works on microstates’ temporal dependencies (Al Zoubi et al., 2019; von Wegner et al., 2017, 2018), this suggests the absence of long-range memory in microstate sequences. Both ALS patients and HC groups showed similar overall AIF content but the contribution of microstate C to the AIF was higher in patients compared with controls (Figure 3).

| 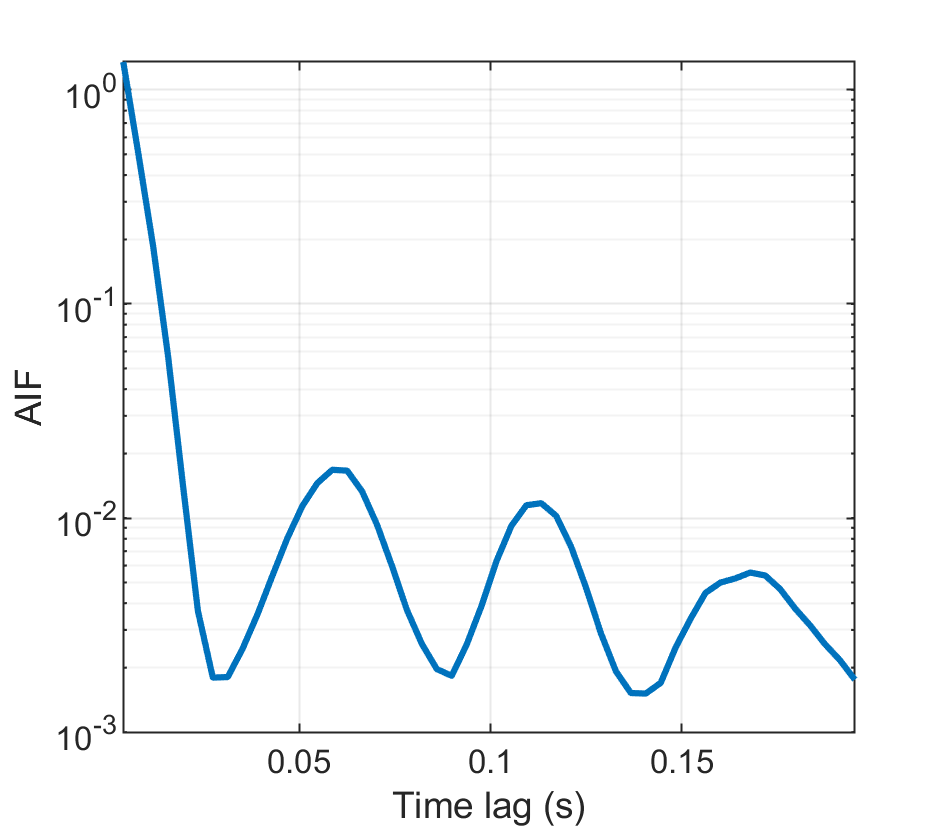  Figure 1: Auto-information or time-lagged mutual information of the microstate sequence for control C47. It shows a periodic oscillation, on a logarithmic y-scale. | 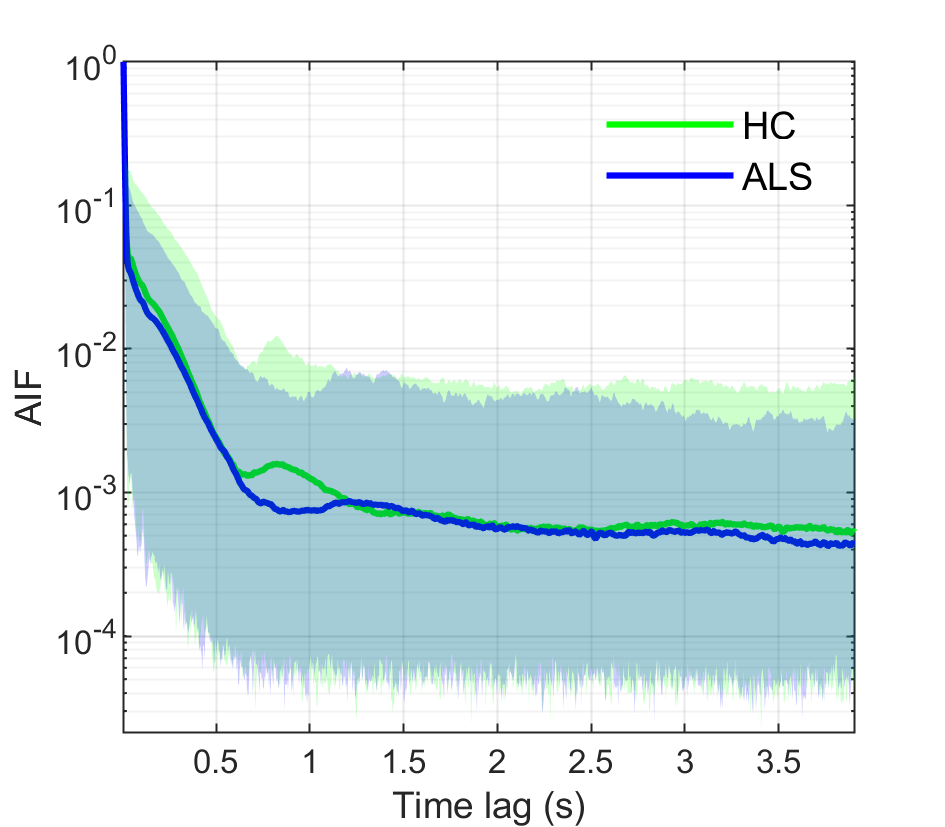  Figure 2: Averaged auto-information of the microstates sequences for HC (green) and ALS patients (blue), with 90% confidence intervals as shaded areas. Logarithmic y-scale. |
| --- | --- |
| 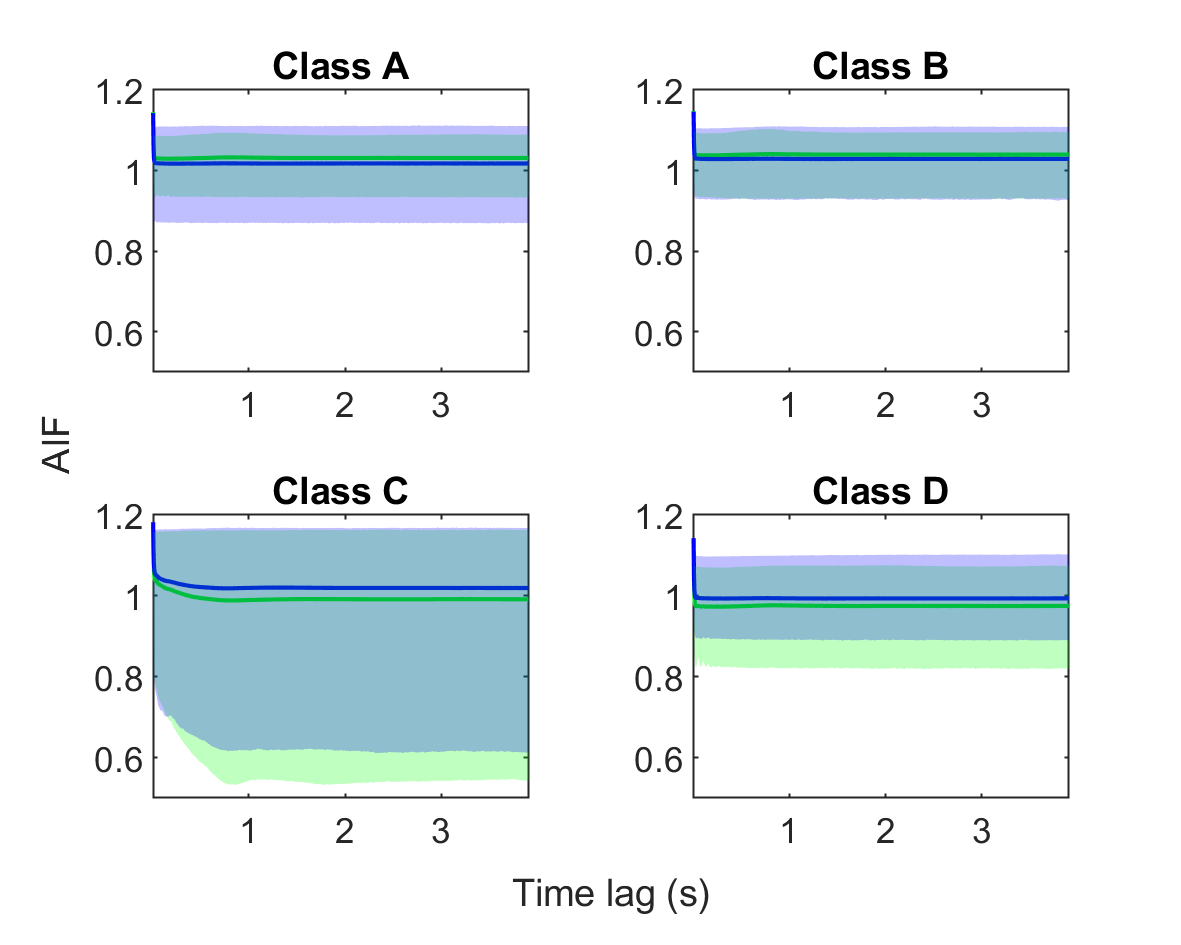  Figure 3: Individual microstate class contributions to the global auto-information function, with 90% confidence intervals as shaded areas, for HC (green) and ALS patients (blue). | |

# Supplementary note 5: Microstate validation step

A K=5 cross-validation analysis was performed on the healthy-controls group. The microstate prototypes were extracted from a portion of the HC and then backfitted on another independent set of HC (test set). No significant difference could be found between cross-validation folds testing sets for any of the four measures of interest: occurrence, coverage, duration, GEV (Kruskal–Wallis one-way ANOVA, p = 0.88).

# References

Abrahams, S., Newton, J., Niven, E., Foley, J., & Bak, T. H. (2014). Screening for cognition and behaviour changes in ALS. *Amyotrophic Lateral Sclerosis and Frontotemporal Degeneration*, *15*(1–2), 9–14. https://doi.org/10.3109/21678421.2013.805784

Al Zoubi, O., Mayeli, A., Tsuchiyagaito, A., Misaki, M., Zotev, V., Refai, H., Paulus, M., Bodurka, J., Investigators, the T. 1000, Aupperle, R. L., Khalsa, S. S., Feinstein, J. S., Savitz, J., Cha, Y.-H., Kuplicki, R., & Victor, T. A. (2019). EEG Microstates Temporal Dynamics Differentiate Individuals with Mood and Anxiety Disorders From Healthy Subjects. *Frontiers in Human Neuroscience*, *13*. https://doi.org/10.3389/fnhum.2019.00056

Cedarbaum, J. M., Stambler, N., Malta, E., Fuller, C., Hilt, D., Thurmond, B., & Nakanishi, A. (1999). The ALSFRS-R: A revised ALS functional rating scale that incorporates assessments of respiratory function. BDNF ALS Study Group (Phase III). *Journal of the Neurological Sciences*, *169*(1–2), 13–21. https://doi.org/10.1016/s0022-510x(99)00210-5

Costello, E., Lonergan, K., Madden, C., O’Sullivan, M., Mays, I., Heverin, M., Pinto-Grau, M., Hardiman, O., & Pender, N. (2020). Equivalency and practice effects of alternative versions of the Edinburgh Cognitive and Behavioral ALS Screen (ECAS). *Amyotrophic Lateral Sclerosis and Frontotemporal Degeneration*, *21*(1–2), 86–91. https://doi.org/10.1080/21678421.2019.1701681

Crockford, C. J., Kleynhans, M., Wilton, E., Radakovic, R., Newton, J., Niven, E. H., Al-Chalabi, A., Hardiman, O., Bak, T. H., & Abrahams, S. (2018). ECAS A-B-C: Alternate forms of the Edinburgh Cognitive and Behavioural ALS Screen. *Amyotrophic Lateral Sclerosis and Frontotemporal Degeneration*, *19*(1–2), 57–64. https://doi.org/10.1080/21678421.2017.1407793

Elamin, M., Pinto-Grau, M., Burke, T., Bede, P., Rooney, J., O’Sullivan, M., Lonergan, K., Kirby, E., Quinlan, E., Breen, N., Vajda, A., Heverin, M., Pender, N., & Hardiman, O. (2017). Identifying behavioural changes in ALS: Validation of the Beaumont Behavioural Inventory (BBI). *Amyotrophic Lateral Sclerosis and Frontotemporal Degeneration*, *18*(1–2), 68–73. https://doi.org/10.1080/21678421.2016.1248976

Pinto-Grau, M., Burke, T., Lonergan, K., McHugh, C., Mays, I., Madden, C., Vajda, A., Heverin, M., Elamin, M., Hardiman, O., & Pender, N. (2017). Screening for cognitive dysfunction in ALS: Validation of the Edinburgh Cognitive and Behavioural ALS Screen (ECAS) using age and education adjusted normative data. *Amyotrophic Lateral Sclerosis and Frontotemporal Degeneration*, *18*(1–2), 99–106. https://doi.org/10.1080/21678421.2016.1249887

Roche, J. C., Rojas-Garcia, R., Scott, K. M., Scotton, W., Ellis, C. E., Burman, R., Wijesekera, L., Turner, M. R., Leigh, P. N., Shaw, C. E., & Al-Chalabi, A. (2012). A proposed staging system for amyotrophic lateral sclerosis. *Brain*, *135*(3), 847–852. https://doi.org/10.1093/brain/awr351

von Wegner, F., Knaut, P., & Laufs, H. (2018). EEG Microstate Sequences From Different Clustering Algorithms Are Information-Theoretically Invariant. *Frontiers in Computational Neuroscience*, *12*, 70. https://doi.org/10.3389/fncom.2018.00070

von Wegner, F., Tagliazucchi, E., & Laufs, H. (2017). Information-theoretical analysis of resting state EEG microstate sequences—Non-Markovianity, non-stationarity and periodicities. *NeuroImage*, *158*, 99–111. https://doi.org/10.1016/j.neuroimage.2017.06.062
